# Supplementary figures and images for: Designing target trials using electronic health records: A case study of second-line disease-modifying anti-rheumatic drugs and cardiovascular disease outcomes in patients with rheumatoid arthritis
Source: PLoS One. 2024 Jun 14;19(6):e0305467. doi: 10.1371/journal.pone.0305467 (PMC11178161; doi:10.1371/journal.pone.0305467)

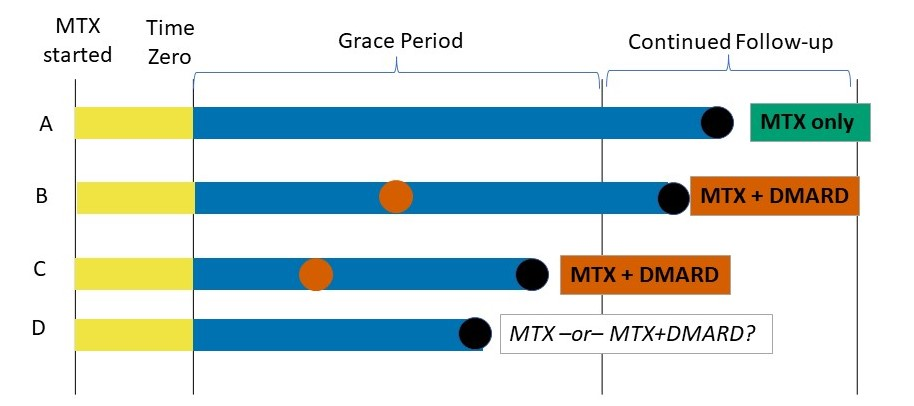

Supplement: S1 Fig — Abbreviations: DMARD–disease-modifying antirheumatic drug, MTX–methotrexate. This figure illustrates data from 4 hypothetical participants. Yellow represents time available in data prior to time zero (not included in analysis). Blue represents follow-up time available in data after time zero. Black circles represent the end of available data for each person (whether an event or censoring). Orange circles represent the initiation of a DMARD prescription. Person A’s data are compatible with the MTX monotherapy strategy, and Person B and C’s data are compatible with the MTX+DMARD strategy. The treatment assignment of individuals like Person D can introduce immortal time bias into analysis, as assigning them all to MTX monotherapy artificially inflates the risk estimates made during the grace period, making MTX monotherapy (possibly incorrectly) appear to be worse than MTX+DMARD. (TIF) [file pone.0305467.s001.tif]

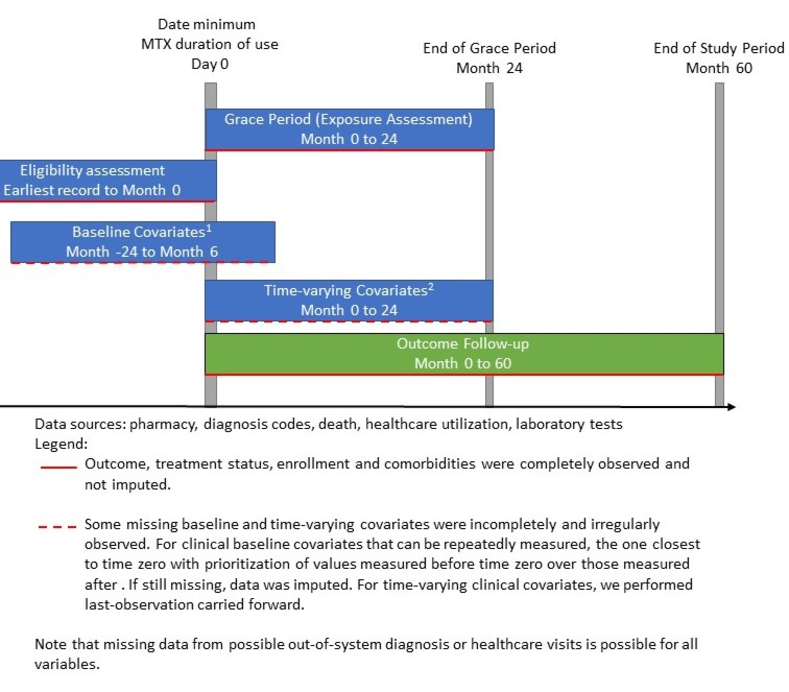

Supplement: S2 Fig — (TIF) [file pone.0305467.s002.tif]

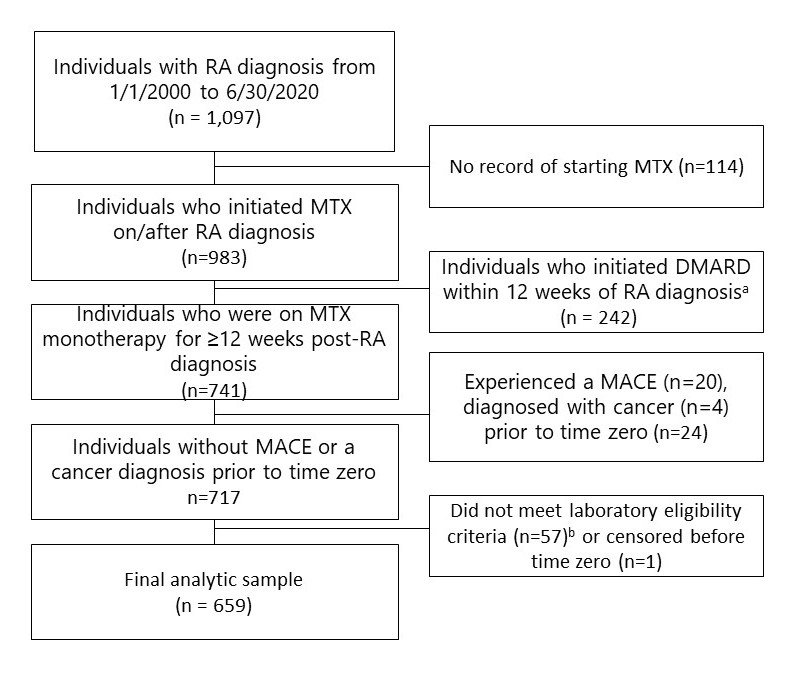

Supplement: S3 Fig — Abbreviations: DMARD–disease-modifying antirheumatic drug, MACE–major adverse cardiac event, MTX–methotrexate, RA–rheumatoid arthritis aIndividuals who initiated a DMARD before time zero were excluded as we could not capture the point in the clinical decision making process when a choice regarding second line therapy was made. bFor laboratory values, we imputed missing baseline laboratory data using random-forest based single imputation before applying the criteria for inclusion. Laboratory eligibility criteria included: Platelet>100,000/mm3, estimated glomerular filtration rate>60 mL/min, White blood cell count>3,000/mm3, Absolute neutrophil count>1200/mm3, Liver transaminases<1.5x upper limit of normal, Hemoglobin>9 g/dL, and Hematocrit>30%. (TIF) [file pone.0305467.s003.tif]

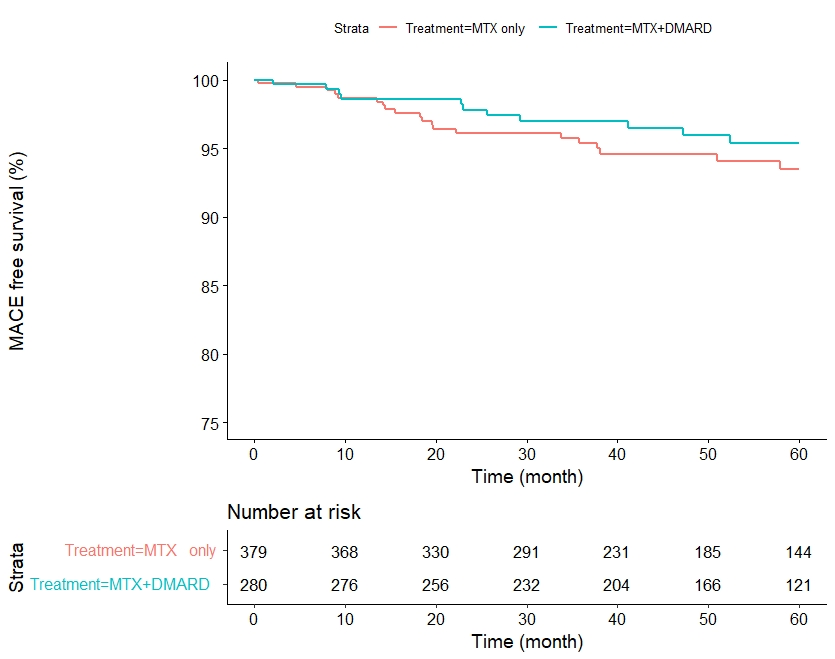

Supplement: S4 Fig — Note: MTX only ‐ only used methotrexate throughout the grace period, MTX+DMARD–added disease-modifying antirheumatic drug to methotrexate at some point during the grace period. (TIF) [file pone.0305467.s004.tif]
