# Supplementary material for: Designing target trials using electronic health records: A case study of second-line disease-modifying anti-rheumatic drugs and cardiovascular disease outcomes in patients with rheumatoid arthritis
Source: PLoS One. 2024 Jun 14;19(6):e0305467. doi: 10.1371/journal.pone.0305467 (PMC11178161; doi:10.1371/journal.pone.0305467)
Supplement: S1 File — (DOCX) [file pone.0305467.s005.docx]

**S1 File: Supplemental Methods and Sample R Code** for

Designing Target Trials Using Electronic Health Records: A Case Study of Second-Line Disease-modifying Anti-Rheumatic Drugs and Cardiovascular Disease Outcomes in Patients with Rheumatoid Arthritis

Short Title: Designing Target Trials in EHRs

Adovich S. Rivera^1,2^

Jacob B. Pierce^3^

Arjun Sinha^4^

Anna E. Pawlowski^5^

Donald M. Lloyd-Jones^4,6^

Yvonne C. Lee^7^

Matthew J. Feinstein^4,6^

Lucia C. Petito^8*^

^1^ Institute for Public Health and Management, Northwestern University Feinberg School of Medicine, Chicago, Illinois, USA

^2^ Department of Research and Evaluation, Kaiser Permanente Southern California, Pasadena, California, USA

^3^ Department of Medicine, Duke University School of Medicine, Durham, North Carolina, USA

^4^ Division of Cardiology, Department of Medicine, Northwestern University Feinberg School of Medicine, Chicago, Illinois, USA

^5^ Northwestern Medicine Enterprise Data Warehouse, Northwestern University, Chicago, Illinois, USA

^6^ Division of Epidemiology, Department of Preventive Medicine, Northwestern University Feinberg School of Medicine, Chicago, Illinois, USA

^7^ Division of Rheumatology, Department of Medicine, Feinberg School of Medicine, Northwestern University, Chicago, Illinois, USA

^8^ Division of Biostatistics, Department of Preventive Medicine, Northwestern University Feinberg School of Medicine, Chicago, Illinois, USA

***Corresponding Author**

**Lucia C. Petito**

Assistant Professor of Preventive Medicine (Biostatistics)

680 N. Lake Shore Drive, 1400-066, Chicago, Illinois, USA, 60611

E-mail address: [lucia.petito@northwestern.edu](mailto:lucia.petito@northwestern.edu)

Phone: 312-503-0985

**Sections**

A. Details on Imputation

B. Sample R Code for Data Analysis

1. **Details on Imputation**

To impute missing baseline covariates, we used single imputation using the missForest package which uses a random forest imputation algorithm to impute data. This was selected over multiple imputation for computational efficiency. Missingness model included demographic covariates, baseline comorbidities, treatment status at end of grace period, outcome status at end of grace period, and all the laboratory variables needed for the eligibility criteria. For lab values, we included values at baseline (closest value before time zero, up to 54 weeks) and values after baseline (value after time zero; up to 24 weeks). Missingness were all in the lab values as follows:

|  | Percent of Final Sample with Missing Data (%) (n = 659) | |
| --- | --- | --- |
|  | Baseline | After baseline |
| Aspartate aminotransferase | 8 | 22 |
| Alanine aminotransferase | 7 | 20 |
| Cholesterol, Total | 70 | 90 |
| Estimated glomerular filtration rate | 8 | 22 |
| Hemoglobin | 7 | 19 |
| Hematocrit | 11 | 23 |
| White blood cell count |  | 19 |

1. **Sample R Code for Data Analysis**

Sample code:

library(missForest)

library(doParallel)

library(doRNG)

#set-up parallel cores

doParallel::registerDoParallel(cores = 4) # set based on number of CPU cores

doRNG::registerDoRNG(seed = 1234)

#clean and set-up wide data for imputation

tab.data$dmard_yn_gp_factor <- as.factor(as.numeric(as.factor(tab.data$dmard_yn_gp)))

tab.data$comp_comorb_b <- as.factor(tab.data$comp_comorb_b)

tab.data$mace_end_gp <- as.factor(tab.data$mace_end_gp)

X <- tab.data %>%

mutate(ins_3cat = ifelse(ins == "private", "pri", ifelse(ins=="Medicaid" | ins=="Medicare", 'gov','none'))) %>%

mutate(ins_3cat = factor(ins_3cat)) %>%

dplyr::select(age_radx_centered, sex, race_eth, ins_3cat, yod_centered, dmard_yn_gp_factor,

htn_b, dm_b, comp_comorb_b,

crcl_bl, crcl_after, crea_bl, crea_after,

wbc_bl, wbc_after, ast_bl, ast_after, alt_bl, alt_after,

hb_bl, hb_after, hct_bl, hct_after,

chol_bl_centered, mace_end_gp)#use mace at end of grace period

#create the imputation object

X.impF <- missForest(X, parallelize = 'forests', verbose=TRUE, mtry=9, maxiter=20, ntree = 128)

#extract imputed dataset

imputed_X <- X.impF$ximp

1. **Sample R Code for Data analysis**

library(tidyverse)

library(plyr)

library(dplyr)

library(lubridate)

library(broom)

library(ggplot2)

library(foreach)

library(rms)

library(tableone)

####I. create functions

##a. create weights using logistic model

create.weights <- function(dflong, wtformN, wtformD, q=0.99){

### Inputs

# dflong - cleaned data in person-time format

# wtformN - numerator formula

# wtformD - denominator formula

# q - upper truncation limit - default is 99.5th percentile

# touseweight - 1 if use row for weight calculation - generally from time zero to grace period

# Weights numerator

wtmodelN <- glm(wtformN, data=dflong[dflong$touseweight==1,], family = quasibinomial())

dflong$pnum <- predict(wtmodelN, newdata = dflong, type='response') # Pr A = 1 | baseline

# Weights denominator

wtmodelD <- glm(wtformD, data=dflong[dflong$touseweight==1,], family = quasibinomial())

dflong$pdenom <- predict(wtmodelD, newdata = dflong, type='response') # Pr A = 1 | baseline, TV

dflong$numCont <- with(dflong, A*pnum + (1-A)*(1-pnum))

dflong$denCont <- with(dflong, A*pdenom + (1-A)*(1-pdenom))

# Numerator

dflong$k1_0 <- with(dflong, ave(numCont, ir_id, FUN=cumprod))

# Denominator

dflong$k1_w <- with(dflong, ave(denCont, ir_id, FUN=cumprod))

# Create both stabilized and unstabilized weights

dflong$stabw <- with(dflong, k1_0 / k1_w)

dflong$unstabw <- with(dflong, 1 / k1_w)

tau <- quantile(dflong$stabw, q)

dflong$stabw.trim <- dflong$stabw

dflong$stabw.trim[dflong$stabw > tau] <- tau

return(dflong)

}

##b. A slightly modified function to create bootstrapped weights

create.weights.boot <- function(dflong, wtformN, wtformD, q=0.99){

#Inputs:

#dflong - cleaned data in person-time format but should be not cloned

# wtformN - numerator formula

# wtformD - denominator formula

# freq - number of times person appeared in bootstrap sample

# q - upper truncation limit - default is 99.5th percentile

# touseweight - 1 if use row for weight calculation - generally from time zero to grace period

# Weights numerator

dflong$pnum <- predict(glm(wtformN, data=dflong[dflong$touseweight==1,], family = quasibinomial(), weights = freq),

newdata = dflong, type='response') # Pr A = 1 | baseline

# Weights denominator

dflong$pdenom <- predict(glm(wtformD, data=dflong[dflong$touseweight==1,], family = quasibinomial(), weights = freq),

newdata = dflong, type='response') # Pr A = 1 | baseline, TV

dflong$numCont <- with(dflong, A*pnum + (1-A)*(1-pnum))

dflong$denCont <- with(dflong, A*pdenom + (1-A)*(1-pdenom))

# Numerator

dflong$k1_0 <- with(dflong, ave(numCont, ir_id, FUN=cumprod))

# Denominator

dflong$k1_w <- with(dflong, ave(denCont, ir_id, FUN=cumprod))

# Create both stabilized and (optional) not stabilized (unstabw) weights

dflong$stabw <- with(dflong, k1_0 / k1_w)

#dflong$unstabw <- with(dflong, 1 / k1_w)

# Make NA all past censoring

dflong$stabw[dflong$touseweight==0] <- NA

#Trim at tau (based on percentile)

tau <- quantile(dflong$stabw, q, na.rm=T)

dflong$stabw[dflong$stabw > tau] <- tau

#trim at 10 (in cases where tau exceeds 10)

dflong$stabw[dflong$stabw > 10] <- 10

#Carry last calculated weight from week 24 forward

dflong <- dflong %>% group_by(ir_id) %>% tidyr::fill(stabw)

return(dflong)

}

dfSet <- data.frame(t_rcs1 = temp$t_rcs1, t_rcs2 = temp$t_rcs2, t_rcs3 = temp$t_rcs3, A=0)

##c. Function for marginal hazards ratio

#This function to does MC simulation to calculate marginal hazard ratios

MCsimHR <- function(i=1, Nsim = 100000, baseline, maxT=72, nind, coeffit, dfSetA0, dfSetA1){

#inputs:

#nind - number of individuals

#coeffit - coefficient of survival model

#dfSetA0 - time data from cloned person-time long data frame but A=0

#dfSetA0 - time data from cloned person-time long data frame but A=1

# First select 100,000 individuals from uncloned data - only keep baseline variables (without A)

set.seed(seeds[i])

extra <- Nsim - nind*floor(Nsim/nind)

ids <- rbind(baseline[rep(1:nind, times = floor(Nsim/nind)),],

dplyr::sample_n(baseline, size=extra, replace=T))

# This completely copies data as many times as possible before reaching Nsim, then samples to create the rest

# Essentially, we are simulating followup floor(Nsim/nind) times for each individual

# Get predictions when A=1

predsA1 <- t(apply(ids, 1, FUN=function(x){

ps <- 1 - 1 / (1 + exp( -1 * as.matrix(suppressWarnings(cbind(1, dfSetA1, t(x)))) %*% coeffit ))

ps[1] <- 1 # Set probability of dying at time 0 to 1

ss <- cumprod(ps)

rbinom(maxT, 1, ss)

}))

# Get predictions when A=0

predsA0 <- t(apply(ids, 1, FUN=function(x){

ps <- 1 - 1 / (1 + exp( -1 * as.matrix(suppressWarnings(cbind(1, dfSetA0, t(x)))) %*% coeffit ))

ps[1] <- 1 # Set probability of dying at time 0 to 1

ss <- cumprod(ps)

rbinom(maxT, 1, ss)

}))

# Identify end of followup for each individual

firstZeroA1 <- apply(predsA1, 1, FUN=function(x){

if (sum(x) == maxT) { return(maxT+1) } else {

return(which.min(x))

}

})

firstZeroA0 <- apply(predsA0, 1, FUN=function(x){

if (sum(x) == maxT) { return(maxT+1) } else {

return(which.min(x))

}

})

# Create data to fit model in

survdf <- data.frame(time = c(firstZeroA1, firstZeroA0),

A = rep(c(1, 0), each=Nsim))

survdf$event <- as.numeric(survdf$time!= (maxT+1)) # Those with maxT+1 survived to the end of follow-up

survdf$time[survdf$time > maxT] <- maxT # Reset those who survived so their maxT is end of follow-up

baseA <- exp(coef(coxph(Surv(time, event) ~ A, data=survdf)))

return(baseA)

}

####II. Set-up data and other parameters

##a. set number of months of GP

gp <- 24

##b. Load clean data files

#tab1.data = wide data frame of only included

#long_fin = long uncloned person-time data frame

#tab1.full = wide data frame of all RA patients

tab1.data <- readRDS("target-tab1data - December 2021.RDS") %>%

filter(t0_dt < censoring_dt) %>% filter(include=="include")

long_fin <- readRDS("target - long_fin - December 2021.RDS") %>%

filter(t0_dt < censoring_dt) %>%

dplyr::select(-clone1_censor_dt, -clone2_censor_dt, -clone1_censor_month, -clone2_censor_month, -idmon.x, -idmon.y)

tab1.full <- tab1.data %>% filter(grp=="full")

##c. Add additional demographics to long_fin

temp <- tab1.data %>% dplyr::select(ir_id, ins_3cat, yod_centered)

long_fin <- merge(long_fin, temp, by="ir_id", all.x=TRUE) %>% group_by(ir_id) %>% dplyr::arrange(t,.by_group=TRUE) %>% distinct(ir_id, t, .keep_all=TRUE) %>%

mutate(ins_pri = ifelse(ins_3cat=="pri", 1, 0), ins_gov = ifelse(ins_3cat=='gov', 1, 0))

##d. Check

length(unique(tab1.data$ir_id)) #659

length(unique(long_fin$ir_id)) #659

with(long_fin, table(mace_yn, t))

#####II. Create long_fin (uncloned long data)

#long_fin is a person-month data set where each eligible individual has t replicates, one for each month from t0 to 60

#make it such that obs is end of observation period

#key variables: A = on DMARD, mace_yn = experience MACE outcome

#touseoutcome = use row for outcome model (max t = 60), touseweight = use row for weights model (max t = 24)

#a. calculate clone 1 censoring month: MTX only during GP; censor if DMARD started during GP

temp <- tab1.full %>%

dplyr::select(ir_id, t0_dt, gp_end_dt, censoring_dt, mace_dt, dt_last_elig_visit, dmard_start_dt) %>%

mutate(gp_end_dt = t0_dt + months(gp))

temp$dmard_start_dt[temp$dmard_start_dt >= temp$gp_end_dt] <- NA #if start DMARD after GP, make it NA so exclude from calc

clone1_dt <- temp %>% rowwise() %>%

dplyr::mutate(clone1_censor_dt = min(c_across(censoring_dt:dmard_start_dt), na.rm=TRUE)) %>%

mutate(clone1_censor_month = floor(( (t0_dt %--% clone1_censor_dt)/ddays(1) )/30) ) %>%

dplyr::select(ir_id, t0_dt, dmard_start_dt, clone1_censor_month, clone1_censor_dt)

clone1_dt <- clone1_dt %>% dplyr::select(ir_id, clone1_censor_month, clone1_censor_dt)

#b. Calculate clone 2 censoring month: start DMARD during GP, censor if does not start DMARD by end of GP

temp <- tab1.full %>%

dplyr::select(ir_id, t0_dt, gp_end_dt, censoring_dt, mace_dt, dt_last_elig_visit, dmard_start_dt) %>%

mutate(gp_end_dt = t0_dt + months(gp))

temp$dmard_start_dt[temp$dmard_start_dt >= temp$gp_end_dt] <- NA #if start DMARD after GP, make it NA so exclude from calc

temp$censoring_dt[is.na(temp$dmard_start_dt)] <- temp$gp_end_dt[is.na(temp$dmard_start_dt)] #effectively, all who didn't start during GP are dropped

temp$censoring_dt[temp$censoring_dt >= ymd("2020-06-30")] <- ymd("2020-06-30") #cap

clone2_dt <- temp %>% rowwise() %>%

dplyr::mutate(clone2_censor_dt = min(c_across(censoring_dt:dt_last_elig_visit), na.rm=TRUE)) %>%

mutate(clone2_censor_month = floor(( (t0_dt %--% clone2_censor_dt)/ddays(1) )/30) ) %>%

dplyr::select(ir_id, t0_dt, dmard_start_dt, clone2_censor_month, clone2_censor_dt)

clone2_dt <- clone2_dt %>% dplyr::select(ir_id, clone2_censor_month, clone2_censor_dt)

#c. add clone1 censoring date to long_fin

long_fin <- merge(long_fin, clone1_dt, by="ir_id", all.x=TRUE) %>% distinct(idmon, .keep_all = TRUE)

#c. add clone2 censoring date to long_fin

long_fin <- merge(long_fin, clone2_dt, by="ir_id", all.x=TRUE) %>% distinct(idmon, .keep_all = TRUE)

length(unique(long_fin$ir_id)) == 659

#d. touse variables

#touseweight - month <= month_obs_gp

#touseweight: 1 = if during grace period (or censored within gp), 0 = no

#to use outcome - month <= months_obs_60

long_fin$touseweight <- ifelse(long_fin$month <= long_fin$months_obs_gp, 1, 0) #month_obs_gp: max month up to end of gp where mace or ltfu has not occurred

long_fin$touseoutcome <- ifelse(long_fin$month <= long_fin$months_obs_60, 1, 0) #month_obs_60: max month up to 60 where mace or ltfu has not occurred

#e. Treatment variable (A)

#A: 1 = on DMARD, 0 = not on DMARD

long_fin <- long_fin %>% mutate(month_dmard_start = floor(((t0_dt %--% dmard_start_dt)/ddays(1))/30) ) #1 month = 30 days

long_fin$A <- ifelse(is.na(long_fin$month_dmard_start), 0, ifelse(long_fin$month >= long_fin$month_dmard_start, 1, 0))

#f. Treatment lag variable (A_lag)

#A_lag = lagged treatment history (6 month lag)

long_fin <- long_fin %>% mutate(A_lag = lag(A,6))

long_fin$A_lag[is.na(long_fin$A_lag)] <- 0

with(long_fin, table(mace_yn, t))

#f. add rcs for time

library(rms)

temp <- as.data.frame(rcspline.eval(long_fin$t, knots=c(3,9,15,21), inclx = TRUE))

names(temp) <- c("t_rcs1", "t_rcs2", "t_rcs3")

long_fin$t_rcs1 <- temp$t_rcs1

long_fin$t_rcs2 <- temp$t_rcs2

long_fin$t_rcs3 <- temp$t_rcs3

####III. Descriptive Statistics

##a. Get DMARD distribution (Supplemental Table 2)

with(tab1.full %>% filter(is.element(ir_id, long_fin$ir_id)), table(dmard_yn_gp, useNA='ifany')) #from creation of long

with(tab1.full %>% filter(is.element(ir_id, long_fin$ir_id)), table(dmard_start, dmard_yn_gp, useNA='ifany')) #from creation of long

temp <- tab1.full %>% filter(is.element(ir_id, long_fin$ir_id)) %>% filter(dmard_yn_gp == "during gp") %>%

mutate(month_to_dmard = (t0_dt %--% dmard_start_dt)/dmonths(1)) %>% dplyr::summarise(n_month_to_dmard=n(),

mn_month_to_dmard=mean(month_to_dmard),

sd_month_to_dmard=sd(month_to_dmard),

md_month_to_dmard=median(month_to_dmard)) %>%

mutate(dmard_start = 'overall', group='overall')

temp2 <- tab1.full %>% filter(is.element(ir_id, long_fin$ir_id)) %>% filter(dmard_yn_gp == "during gp") %>%

mutate(month_to_dmard = (t0_dt %--% dmard_start_dt)/dmonths(1)) %>%

group_by(dmard_start) %>% dplyr::summarise(n_month_to_dmard=n(),

mn_month_to_dmard=mean(month_to_dmard),

sd_month_to_dmard=sd(month_to_dmard),

md_month_to_dmard=median(month_to_dmard)) %>% mutate(group='drug')

temp3 <- tab1.full %>% filter(is.element(ir_id, long_fin$ir_id)) %>% filter(dmard_yn_gp == "during gp") %>%

mutate(dmard_start = mapvalues(dmard_start, sort(unique(tab1.full$dmard_start[tab1.full$dmard_yn_gp=="during gp"])),

c("abatacept", 'adalimumab', 'others', 'others', 'etanercept', 'others',

'hydroxychloroquine', 'infliximab', 'leflunomide', 'others', 'others', 'sulfasalazine',

'others', 'tofacitinib', 'others'))) %>%

mutate(month_to_dmard = (t0_dt %--% dmard_start_dt)/dmonths(1)) %>%

group_by(dmard_start) %>% dplyr::summarise(n_month_to_dmard=n(),

mn_month_to_dmard=mean(month_to_dmard),

sd_month_to_dmard=sd(month_to_dmard),

md_month_to_dmard=median(month_to_dmard)) %>% mutate(group='reduced')

write.csv(rbind(temp, temp2, temp3), "target - first dmard - December 2021.csv")

##b. Table 1

vars <- c("age_t0", "sex", "race_4cat", "year_t0",

"htn_yn0", "dm_yn0", "comp_comorb_yn0", "gfr0", "chol0",

"htn_yn", "dm_yn", "comp_comorb_yn", "gfr", "chol", "ins_3cat", "yod_centered")

catvars <- c("sex", "race_4cat",

"htn_yn0", "dm_yn0", "comp_comorb_yn0",

"htn_yn", "dm_yn", "comp_comorb_yn","ins_3cat")

#at time zero

tab.data <- long_fin %>% filter(t==0) %>% #mace_yn now allowed

mutate(year_t0 = year(t0_dt))

nrow(tab.data) ==659 #must be true

tab1 <- CreateTableOne(vars=vars, factorVars = catvars, data = tab.data)

write.csv(print(tab1),"target - tab1 - baseline - December 2021.csv")

tab1st <- CreateTableOne(vars=vars, factorVars = catvars, data = tab.data, strata='dmard_yn_gp')

write.csv(print(tab1st),"target - tab1 - baseline - st - December 2021.csv")

#at end of GP

tab.data <- long_fin %>% group_by(ir_id) %>% mutate(mace_yn_lag = lag(mace_yn)) %>%

filter(t==gp) %>% filter(mace_yn_lag==0) %>%

mutate(year_t0 = year(t0_dt))

dim(tab.data)

tab1 <- CreateTableOne(vars=vars, factorVars = catvars, data = tab.data)

write.csv(print(tab1),"target - tab1 - end of gp - December 2021.csv")

tab1st <- CreateTableOne(vars=vars, factorVars = catvars, data = tab.data, strata='A')

write.csv(print(tab1st),"target - tab1 - end of gp - st - December 2021.csv")

#at end of 60months

temp <- long_fin %>% group_by(ir_id) %>% mutate(mace_yn_lag = lag(mace_yn)) %>%

filter(t==gp) %>% filter(mace_yn_lag==0)

tab.data <- long_fin %>% filter(is.element(ir_id, temp$ir_id)) %>% filter(t==60)

table(tab.data$mace_yn)

with(tab.data, table(A,mace_yn))

#####IV. Naive analysis

library(survival)

library(survminer)

##a. Set-up data and calculate number of events

temp <- tab1.full %>% dplyr::rowwise() %>%

dplyr::mutate(Time_censor_dt = min(censoring_dt, dt_last_elig_visit, na.rm=TRUE))

temp$Time_event_dt <- temp$Time_censor_dt

temp$Time_event_dt[!is.na(temp$mace_dt) & temp$mace_dt <= temp$Time_censor_dt] <- temp$mace_dt[!is.na(temp$mace_dt) & temp$mace_dt <= temp$Time_censor_dt]

head(temp[c("censoring_dt", "dt_last_elig_visit", "gp_end_dt", "mace_dt", "Time_event_dt", "Time_censor_dt")])

temp <- temp %>% mutate(Time = (t0_dt %--% Time_event_dt)/dmonths(1),

Event = ifelse(!is.na(mace_dt) & mace_dt <= Time_censor_dt, 1, 0)) %>%

mutate(Event = ifelse(Time > 60, 0, Event)) %>%

mutate(Time = ifelse(Time > 60, 60, Time),

dmard_yn = ifelse(is.na(dmard_start_dt), 0, ifelse(dmard_start_dt <= gp_end_dt, 1, 0)))

temp2 <- long_fin %>% filter(t==0) %>% dplyr::select(ir_id, age_t0, sex, re_black, re_hisp, re_other,

gfr0, chol0, dm_yn0, htn_yn0, comp_comorb_yn0)

temp <- merge(temp, temp2, by="ir_id", all.x=TRUE) %>% distinct(ir_id, .keep_all=TRUE)

table(temp$Event, useNA='ifany') #should be 31

##b. Unweighted, noncloned, and unadjusted cox

fit<- survfit(Surv(Time, Event) ~ dmard_yn, data = temp)

ggsurvplot(fit, censor=FALSE, ylim=c(0.75,1))

res <- coxph(Surv(Time, Event) ~ dmard_yn, data = temp)

tidy(res, exp=TRUE, conf.int=TRUE)

##c. Unweighted, noncloned, and adjusted cox

res <- coxph(Surv(Time, Event) ~ dmard_yn + age_t0 + sex.y + re_black + re_hisp + re_other + gfr0 + chol0 +

dm_yn0 + htn_yn0 + comp_comorb_yn0 + ins_3cat + yod_centered, data = temp)

tidy(res, exp=TRUE, conf.int=TRUE)

#####V. Calculate Weights using uncloned data (long_fin)

##a. Calculate weights

df <- long_fin %>% mutate(t = month) %>% filter(t <= gp) %>% filter(touseweight==1)

wtformN <- A ~ t_rcs1 + t_rcs2 + t_rcs3 + A_lag + age_t0 + sex + re_black + re_hisp + re_other +

gfr0 + chol0 + dm_yn0 + htn_yn0 + comp_comorb_yn0 + ins_pri + ins_gov + yod_centered

wtformD <- A ~ t_rcs1 + t_rcs2 + t_rcs3 + A_lag + age_t0 + sex + re_black + re_hisp + re_other +

gfr0 + chol0 + dm_yn0 + htn_yn0 + comp_comorb_yn0 + ins_pri + ins_gov + yod_centered +

gfr + chol + dm_yn + htn_yn + comp_comorb_yn

df2 <- create.weights(df, wtformN, wtformD, q=0.995)

temp1 <- df2 %>% group_by(ir_id) %>% slice(which.max(t)) %>% ungroup() #get last weight per person

temp2 <- long_fin %>% filter(touseweight==0) %>% #get obs after gp/censoring

mutate(pnum=NA, pdenom=NA, numCont=NA, denCont=NA, k1_0=NA, k1_w=NA, stabw=NA, unstabw=NA, stabw.trim=NA)

df3 <- rbind(temp1, temp2) %>% group_by(ir_id) %>% dplyr::arrange(t, .by_group=TRUE) %>%

tidyr::fill(stabw, stabw.trim, unstabw) %>% filter(touseweight==0) #fill stabilized weights

summary(df3$stabw.trim) #no NA

summary(df3$stabw.trim[df3$t==24]) #no NA

ggplot(df3 %>% filter(t==24)) +

geom_violin(aes(x=factor(A), y=stabw.trim)) #good overlap

#####VI. Calculate outcome model using cloned data

##a. Create cloned outcome data frame (outcome.df) from uncloned (long_fin_outcome)

long_fin_outcome <- rbind(df2, df3) %>% group_by(ir_id) %>% dplyr::arrange(t, .by_group=TRUE) %>%

distinct(ir_id, month, .keep_all=TRUE) %>% ungroup()

#table(long_fin_outcome$month, long_fin_outcome$mace_yn)

clone1 <- long_fin_outcome %>% filter(month <= clone1_censor_month) %>% mutate(clone=0) #mtx only clone

clone2 <- long_fin_outcome %>% filter(month <= clone2_censor_month) %>% mutate(clone=1) #start dmard during gp clone

outcome.df <- rbind(clone1, clone2) %>% mutate(clonet = clone*t, clonet2 = clone*t*t)

##b. Set-up formulas

hrform <- mace_yn ~ t_rcs1 + t_rcs2 + t_rcs3 + clone +

age_t0 + sex + re_black + re_hisp + re_other + gfr0 + chol0 +

dm_yn0 + htn_yn0 + comp_comorb_yn0 + ins_pri + ins_gov + yod_centered

ixform <- mace_yn ~ t_rcs1 + t_rcs2 + t_rcs3 + clone +

age_t0 + sex + re_black + re_hisp + re_other + gfr0 + chol0 +

dm_yn0 + htn_yn0 + comp_comorb_yn0 + ins_pri + ins_gov + yod_centered + gfr + chol + dm_yn + htn_yn + comp_comorb_yn

hrform.SA <- mace_yn ~ t_rcs1*clone + t_rcs2*clone + t_rcs3*clone +

age_t0 + sex + re_black + re_hisp + re_other + gfr0 + chol0 +

dm_yn0 + htn_yn0 + comp_comorb_yn0 + ins_pri + ins_gov + yod_centered

ixform.SA <- mace_yn ~ t_rcs1*clone + t_rcs2*clone + t_rcs3*clone +

age_t0 + sex + re_black + re_hisp + re_other + gfr0 + chol0 +

dm_yn0 + htn_yn0 + comp_comorb_yn0 + ins_pri + ins_gov + yod_centered + gfr + chol + dm_yn + htn_yn + comp_comorb_yn

##c. models

#Unadjusted unweighted

plrfit <- glm(mace_yn ~ t_rcs1 + t_rcs2 + t_rcs3 + clone,

data = outcome.df[outcome.df$touseoutcome==1,], family=quasibinomial())

#Adjusted unweighted

adjplrfit <- glm(hrform,

data = outcome.df[outcome.df$touseoutcome==1,], family=binomial())

#Adjusted ix form unweighted

adjplrixfit <- glm(ixform,

data = outcome.df[outcome.df$touseoutcome==1,], family=binomial())

#Unadjusted weighted

wplrfit <- glm(mace_yn ~ t_rcs1 + t_rcs2 + t_rcs3 + clone,

data = outcome.df[outcome.df$touseoutcome==1,], weights = stabw.trim, family=quasibinomial())

#Adjusted weighted

wadjplrfit <- glm(hrform,

data = outcome.df[outcome.df$touseoutcome==1,], weights = stabw.trim, family=quasibinomial())

#Adjusted ix form weighted

wadjplrixfit <- glm(ixform,

data = outcome.df[outcome.df$touseoutcome==1,], weights = stabw.trim, family=quasibinomial())

#Adjusted with interaction

wadjplrfit.SA <- glm(hrform.SA,

data = outcome.df[outcome.df$touseoutcome==1,], weights = stabw.trim, family=quasibinomial())

wadjplrixfit.SA <- glm(ixform.SA,

data = outcome.df[outcome.df$touseoutcome==1,], family=binomial())

##d. compile results

res1 <- tidy(plrfit, exp=TRUE, conf.int=TRUE) %>% mutate(model="unadj unw", adj=0, wt=0, td=0, int=0)

res2 <- tidy(adjplrfit, exp=TRUE, conf.int=TRUE) %>% mutate(model="adj unw", adj=1, wt=0, td=0, int=0)

res3 <- tidy(adjplrixfit, exp=TRUE, conf.int=TRUE) %>% mutate(model="adj unw ix", adj=1, wt=0, td=1, int=0)

res4 <- tidy(wplrfit, exp=TRUE, conf.int=TRUE) %>% mutate(model="unadj w", adj=0, wt=1, td=0, int=0)

res5 <- tidy(wadjplrfit, exp=TRUE, conf.int=TRUE) %>% mutate(model="adj w", adj=1, wt=1, td=0, int=0)

res6 <- tidy(wadjplrixfit, exp=TRUE, conf.int=TRUE) %>% mutate(model="adj w ix", adj=1, wt=1, td=1, int=0)

res7 <- tidy(wadjplrfit.SA, exp=TRUE, conf.int=TRUE) %>% mutate(model="adj w SA", adj=1, wt=1, td=0, int=1)

res8 <- tidy(wadjplrixfit.SA, exp=TRUE, conf.int=TRUE) %>% mutate(model="adj w ix SA", adj=1, wt=1, td=1, int=1)

res.fin <- rbind(res1, res2, res3, res4, res5, res6, res7, res8)

res.clone <- res.fin %>% filter(term=="clone")

write.csv(res.clone, "target - main - December 2021.csv") #results for the weighted analysis

#####VII. Calculate Survival curves with bootstrap (Figure 2)

###A. calculate original survival curve

#a. create model

hrform <- mace_yn ~ t_rcs1 + t_rcs2 + t_rcs3 + clone +

age_t0 + sex + re_black + re_hisp + re_other + gfr0 + chol0 +

dm_yn0 + htn_yn0 + comp_comorb_yn0 + ins_pri + ins_gov + yod_centered

wadjplrixfit <- glm(hrform,

data = outcome.df[outcome.df$touseoutcome==1,], weights = stabw.trim, family=quasibinomial())

#b. make clones for survival curve df - all individuals from month 0 to 60 will contribute

temp <- long_fin_outcome %>% mutate(clone=0) #mtx only clone

temp2 <- long_fin_outcome %>% mutate(clone=1) #start dmard during gp clone

surv.df <- rbind(temp, temp2) %>% mutate(clonetr1 = clone*t_rcs1, clonetr2 = clone*t_rcs2, clonetr3 = clone*t_rcs3)

#b. Calculate probability of survival in MTX only clones (clone here will include month 0 to month 60)

mtxonly <- surv.df[surv.df$clone==0,]

mtxonly$p <- 1 - predict(wadjplrfit, newdata=mtxonly, type='response')

mtxonly$s <- with(mtxonly, ave(p, ir_id, FUN=cumprod))

#c. Calculate probability of survival With DMARDS clones (clone here will include month 0 to month 60)

withdmard <- surv.df[surv.df$clone==1,]

withdmard$p <- 1 - predict(wadjplrfit, newdata=withdmard, type='response')

withdmard$s <- with(withdmard, ave(p, ir_id, FUN=cumprod))

both <- rbind(mtxonly, withdmard)

both <- both[,c('s', 'clone', 't')]

#d. Calculate the mean survival at each visit within each treatment arm

results.org <- aggregate(s ~ t + clone, FUN=mean, data=both) %>%

dplyr::rename(treatedf=clone) %>%

mutate(boot=0, treatedf=ifelse(treatedf==1,"Start DMARD", "No DMARD"),

t = t + 1)

temp <- data.frame("t"=c(0,0), "s" = c(1,1), boot=0,

"treatedf"= c("No DMARD", "Start DMARD"))

results.org <- rbind(results.org, temp) %>% dplyr::arrange(t, treatedf)

with(surv.df, table(mace_yn, t)) #event at t0 allowed coz it's event is Y at t+1 (e.g. Y at 1 for t=0)

head(results.org) #should have s = 1 at t = 0

#e. plot the survival curve

ggplot(data = results.org) +

geom_line(aes(y=s, x=t, group=treatedf, color=factor(treatedf))) +

theme_bw()

###B. Calculate the bootstrap survival

#treatment weights capped at 10

#a. Get bootstrap sample of ids

df <- long_fin_outcome

set.seed(123)

B=500 # Number of bootstrap reps (500)

ids <- unique(df$ir_id) # Unique IDs

nind <- length(ids) # Number of individuals

bootids1 <- matrix(ids[sample(nind, nind*B, replace=T)], nrow=nind, ncol=B) # This is a matrix that has B columns, with the n resampled IDs already selected

bootids2 <- apply(bootids1, 2, FUN=function(b){

temp <- data.frame(ir_id = b)

return(plyr::count(temp, vars = 'ir_id'))

}) # This returns a matrix with 2 variables: the ID for each unique individual ('ir_id'), and the frequency they appeared in a particular bootstrap sample ('freq')

#b. Calculate bootstrap mean survival at each visit

df <- long_fin_outcome #not cloned data

results.fin <- data.frame("t"=NA, "treatedf"=NA, 's'=NA, 'boot'=NA)

bootres3 <- for(i in 1:B){

# Load libraries

require(reshape2)

require(plyr)

require(dplyr)

require(rms)

#1. create uncloned with frequency weights (based on how often id was sampled)

bootdf <- left_join(df, bootids2[[i]], by = "ir_id")

bootdf$freq[is.na(bootdf$freq)] <- 0

#2. calculate weights in bootstrap sample (max weight of 10)

bootdf <- create.weights.boot(bootdf, wtformN, wtformD, q=0.99)

#3. clone the data

clone1 <- bootdf %>% filter(month <= clone1_censor_month) %>% mutate(clone=0) #mtx only clone

clone2 <- bootdf %>% filter(month <= clone2_censor_month) %>% mutate(clone=1) #start dmard during gp clone

boot.cloned.df <- rbind(clone1, clone2) #for weights model and outcome

temp1 <- bootdf %>% mutate(clone=0) #mtx only clone

temp2 <- bootdf %>% mutate(clone=1) #start dmard during gp clone

boot.surv.df <- rbind(temp1, temp2) #for survival plot

#4. calculate outcome model

if(sum(is.na(boot.cloned.df$stabw.trim)) != 0){

results.boot <- data.frame("t"=777, "treatedf"=NA, 's'=NA, 'boot'=i)

} else {

model <- glm(hrform, data = boot.cloned.df[boot.cloned.df$touseoutcome==1 & boot.cloned.df$freq >0,],

weights = stabw.trim*freq, family=quasibinomial())

}

if (model$converged == TRUE){

#5.calculate s per clone (should I account for repeated sampling in bootstrap?)

#clones should include full obs from 0 to 60

#see: https://github.com/eleanormurray/CausalSurvivalAnalysisWorkshop/blob/master/R/workshop_v6_tidy.R

# In MTX only clones

mtxonly <- boot.surv.df[boot.surv.df$clone==0,] %>% filter(freq!=0)

mtxonly$p <- 1 - predict(model, newdata=mtxonly, type='response')

mtxonly$s <- with(mtxonly, ave(p, ir_id, FUN=cumprod))

# With DMARDS clones

withdmard <- boot.surv.df[boot.surv.df$clone==1,] %>% filter(freq!=0)

withdmard$p <- 1 - predict(model, newdata=withdmard, type='response')

withdmard$s <- with(withdmard, ave(p, ir_id, FUN=cumprod)) #double-check: every person, calculate cum prob at each time and then take average for each time

#6. Calculate the mean survival at each visit within each treatment arm

both <- rbind(mtxonly, withdmard)

both <- both[,c('s', 'clone', 't', 'freq')]

results.boot <- both %>% filter(freq!=0) %>%

group_by(clone, t) %>% dplyr::summarise(s = weighted.mean(s,freq), .groups='keep') %>%

mutate(boot=i, clone = ifelse(clone==1, "Start DMARD", "No DMARD"), t=t+1) %>%

dplyr::rename(treatedf = clone) %>% dplyr::select("t", "treatedf", "s", "boot") %>% ungroup()

temp <- data.frame("t"=c(0,0), "s" = c(1,1), boot=i,

"treatedf"= c("No DMARD", "Start DMARD"))

results.boot <- rbind(results.boot, temp) %>% dplyr::arrange(t, treatedf)

} else {

results.boot <- data.frame("t"=999, "treatedf"=NA, 's'=NA, 'boot'=i)

}

#8. bind results

results.fin <- rbind(results.fin, results.boot) %>% filter(!is.na(t))

}

sc.bs.res <- results.fin

write.csv(rbind(results.org, sc.bs.res), "target - surv curve - main - December 2021.csv")

#c. inspect results for nonconvergence (777) or negative weights (999)

with(results.fin, table(boot, t>=777))

#d. plot survival curves

plot.data <- read.csv("target - surv curve - main - December 2021.csv") %>% mutate(orig = ifelse(boot==0, 1, 0)) %>%

filter(t < 777) %>% mutate(boot_t = paste0(treatedf,boot))

ggplot(plot.data) +

geom_line(aes(x = t, y = s, group=rev(boot), col=factor(orig))) +

facet_grid(~treatedf)

head(plot.data)

#e. get 95%ci for survival for each time point

bs.sc.res <- plot.data %>% filter(orig==0) %>% group_by(t, treatedf) %>%

dplyr::summarise(p05=quantile(s,0.025), md=quantile(s,0.5), p975=quantile(s,0.975))

bs.sc.res %>% filter(t==0)

bs.sc.res %>% filter(t==24)

bs.sc.res %>% filter(t==60)

#f. survival curves can be used to calculate risk differences

x= bs.sc.res #survival curves

time = 60 #change as needed

#main results

sA0.main <- (x %>% filter(boot==0 & t==time & treatedf=="No DMARD"))$s

sA1.main <- (x %>% filter(boot==0 & t==time & treatedf=="Start DMARD"))$s

rA0.main <- 1-sA0.main

rA1.main <- 1-sA1.main

rd.main = (rA1.main - rA0.main)*100

#bootstrapped results

rd.60 <- NA

for (i in 1:max(x$boot)){

sA0 <- (x %>% filter(boot==i & t==time & treatedf=="No DMARD"))$s

sA1 <- (x %>% filter(boot==i & t==time & treatedf=="Start DMARD"))$s

rA0 = 1-sA0 #inverse MACE-free survival to risk

rA1 = 1-sA1 #inverse MACE-free survival to risk

rd <- rA1-rA0

rd.60 <- rbind(rd.60, rd)

}

#final results for risk difference (rd.res)

rd.res <- data.frame(

t=time,

sa0.per=sA0.main*100,

sa1.per=sA1.main*100,

ra0.per=rA0.main*100,

ra1.per=rA1.main*100,

rd.per = rd.main,

rd.per.ll = quantile(rd.60, na.rm=TRUE, c(0.025))*100,

rd.per.ul = quantile(rd.60, na.rm=TRUE, c(0.975))*100) %>%

mutate(rd_ci = paste0(round(rd.per,2)," (",round(rd.per.ll,2),", ",

round(rd.per.ul,2),")"))

#f. survival curves can be used to calculate restricted mean survival time

rmst_time = 60 #change as needed

x= bs.sc.res #survival curves

#function to get RMST given survival curves and time

get_RMST <- function(data, time=15){

require(MESS)

temp <- data %>% filter(treatedf=="Start DMARD") %>% filter(t<=time)

rmst_A1 <- auc(temp$t, temp$s, na.rm=TRUE)

temp <- data %>% filter(treatedf=="No DMARD") %>% filter(t<=time)

rmst_A0 <- auc(temp$t, temp$s, na.rm=TRUE)

rmst_diff <- rmst_A1 - rmst_A0

res <- data.frame("rmst_A1" = rmst_A1, "rmst_A0" = rmst_A0, "rmst_diff" = rmst_diff)

return(res)

}

#calculate RMST for bootstrap sc

rmst_res <- get_RMST(x %>% filter(boot==0), time=rmst_time) %>% mutate(boot=0)

for(i in 1:max(x$boot)){

temp <- x %>% filter(boot==i)

res <- get_RMST(temp, time=rmst_time) %>% mutate(boot=i)

rmst_res <- rbind(rmst_res, res)

}

rmst_res %>% filter(boot==0)

quantile(rmst_res$rmst_diff, p=c(0.025, 0.5, 0.975))

#####VII. standardized Monte Carlo hazard ratio

library(doParallel)

library(foreach)

#################FUNCTIONS FOR MARGINAL HR

#A slightly modified function to create bootstrapped weights - add frequency weights

create.weights.boot <- function(dflong, wtformN, wtformD, q=0.99){ #dflong should be not cloned

#fitN <- glm(wtformN, data=dflong[dflong$touseweight==1,], family = quasibinomial(), weights = freq)

dflong$pnum <- predict(glm(wtformN, data=dflong[dflong$touseweight==1,], family = quasibinomial(), weights = freq),

newdata = dflong, type='response') # Pr A = 1 | baseline

# Weights denominator

dflong$pdenom <- predict(glm(wtformD, data=dflong[dflong$touseweight==1,], family = quasibinomial(), weights = freq),

newdata = dflong, type='response') # Pr A = 1 | baseline, TV

dflong$numCont <- with(dflong, A*pnum + (1-A)*(1-pnum))

dflong$denCont <- with(dflong, A*pdenom + (1-A)*(1-pdenom))

# Set contribution at baseline to 1

dflong$numCont[dflong$t %in% c(0)] <- 1

dflong$denCont[dflong$t %in% c(0)] <- 1

# Numerator

dflong$k1_0 <- with(dflong, ave(numCont, ir_id, FUN=cumprod))

# Denominator

dflong$k1_w <- with(dflong, ave(denCont, ir_id, FUN=cumprod))

# Create both stabilized and unstabilized weights

dflong$stabw <- with(dflong, k1_0 / k1_w)

#dflong$unstabw <- with(dflong, 1 / k1_w)

# Make NA all past censoring

dflong$stabw[dflong$touseweight==0] <- NA

#Trim at tau (based on percentile)

tau <- quantile(dflong$stabw, q, na.rm=T)

dflong$stabw[dflong$stabw > tau] <- tau

#trim at 10

dflong$stabw[dflong$stabw > 10] <- 10

#loocf from week 24

dflong <- dflong %>% group_by(ir_id) %>% tidyr::fill(stabw)

return(dflong)

}

#########Calculate marginal HR

hrform <- mace_yn ~ t_rcs1 + t_rcs2 + t_rcs3 + A + age_t0 + sex +

re_black + re_hisp + re_other + gfr0 + chol0 +

dm_yn0 + htn_yn0 + comp_comorb_yn0 + ins_pri + ins_gov + yod_centered

basecoef <- coef(adjplrfit) # Estimates from adjusted unweighted hr model

mcmc.df <- outcome.df

hr.data <- outcome.df[outcome.df$touseoutcome==1,]

adjplrfit <- glm(hrform,

data = hr.data, family=binomial())

#a. set-up models and save coefficients - run outcomes model using cloned data

mcmc.df <- hr.data

mcmc.df$sex <- ifelse(mcmc.df$sex=="M", 1, 0)

hrform <- mace_yn ~ t_rcs1 + t_rcs2 + t_rcs3 + A + age_t0 + sex +

re_black + re_hisp + re_other + gfr0 + chol0 +

dm_yn0 + htn_yn0 + comp_comorb_yn0 + ins_pri + ins_gov + yod_centered

adjplrfit <- glm(hrform,

data = mcmc.df[mcmc.df$touseoutcome==1,], family=binomial()) #use cloned coz it's an outcome model

basecoef <- coef(adjplrfit) # Estimates from adjusted unweighted hr model

wcoef <- coef(glm(hrform,

data = mcmc.df[mcmc.df$touseoutcome==1,],

weights = stabw, family=quasibinomial()) )

#b. Set-up Baseline dataset - keeps original people only (uncloned)

df <- mcmc.df[mcmc.df$touseoutcome==1,]

baseline <- as.data.frame(df[df$t==0 & df$touseweight==1,] ) #the touseweight makes this uncloned

# IMPORTANT to keep variables in order from model fits

baseline <- baseline[,c("age_t0", "sex", "re_black", "re_hisp", "re_other", "gfr0", "chol0", "dm_yn0", "htn_yn0", "comp_comorb_yn0", "ins_pri", "ins_gov", "yod_centered")]

#c. Code to do MC simulation to calculate marginal hazard ratios + bootstrap CIs

set.seed(1)

B <- 501 # Needs to be number of times plus 1

maxT <- 60

seeds <- sample.int(100000000, size=B)

# Set up treatment, time, and interaction variables

temp <- hr.data %>% ungroup() %>% dplyr::select(t, t_rcs1, t_rcs2, t_rcs3) %>% distinct(.)

head(temp)

t <- 0:maxT

dfSetA0 <- data.frame(t_rcs1 = temp$t_rcs1, t_rcs2 = temp$t_rcs2, t_rcs3 = temp$t_rcs3, A=0)

dfSetA1 <- data.frame(t_rcs1 = temp$t_rcs1, t_rcs2 = temp$t_rcs2, t_rcs3 = temp$t_rcs3, A=1)

margHR.uw <- MCsimHR(i=1, Nsim = 1000, nind=660, baseline, maxT=60, coeffit=basecoef, dfSetA0, dfSetA1)

margHR.w <- MCsimHR(i=1, Nsim = 1000, nind=660, baseline, maxT=60, coeffit=wcoef, dfSetA0, dfSetA1)

margHR.w

###############

registerDoParallel(cores=detectCores() - 1)

getDoParWorkers()

set.seed(12345)

B <- 501

ids <- unique(mcmc.df$ir_id) #mcmc.df is a cloned dataset but can also used uncloned for this line

nind <- length(ids)

bootids1 <- matrix(ids[sample.int(nind, nind*B, replace=T)], nrow=nind, ncol=B)

bootids2 <- apply(bootids1, 2, FUN=function(b){

temp <- data.frame(ir_id = b)

return(plyr::count(temp, vars = 'ir_id'))

}) # This returns a matrix with 2 variables: the ID for each unique individual ('ir_id'), and the frequency they appeared in a particular bootstrap sample ('freq')

#Bootstrap loop

#gives marginal HR at baseline and Time-varyin using hrform

hrform <- mace_yn ~ t_rcs1 + t_rcs2 + t_rcs3 + clone + age_t0 + sex +

re_black + re_hisp + re_other + gfr0 + chol0 +

dm_yn0 + htn_yn0 + comp_comorb_yn0 + ins_pri + ins_gov + yod_centered

mhr.bootres.test2 <- foreach(i=2:B, .combine=rbind) %dopar% {

# Load libraries

require(foreach)

require(reshape2)

require(plyr)

require(dplyr)

require(rms)

print(i)

#1. Add "freq" variable to boot

df <- long_fin #df is uncloned dataframe

df$sex <- ifelse(df$sex=="M", 1, 0)

bootdf <- left_join(df, bootids2[[i]], by = "ir_id")

bootdf$freq[is.na(bootdf$freq)] <- 0

#2. Create weights - using uncloned data and includes freq as a weight

bootdf <- create.weights.boot(bootdf, wtformN, wtformD, q=0.99) #uses uncloned with weights - uses freq as a weight in the model

#3. clone the data

clone1 <- bootdf %>% filter(month <= clone1_censor_month) %>% group_by(ir_id) %>%

dplyr::arrange(month, .by_group = TRUE) %>% mutate(clone=0) %>% ungroup()#mtx only clone

clone2 <- bootdf %>% filter(month <= clone2_censor_month) %>% group_by(ir_id) %>%

dplyr::arrange(month, .by_group = TRUE) %>% mutate(clone=1) %>% ungroup() #start dmard during gp clone

boot.cloned.df <- rbind(clone1, clone2) #for outcome model

#3. Get model coefficients from outcomes model - WEIGHTED and CLONED

wcoef <- coef(glm(hrform,

data = boot.cloned.df[boot.cloned.df$touseoutcome==1 & boot.cloned.df$freq>0,],#exclude freq=0

weights = stabw*freq, family=quasibinomial()) )

# Get model from outcomes model coefficients - UNWEIGHTED and CLONED

bcoef <- coef(glm(hrform,

data = boot.cloned.df[boot.cloned.df$touseoutcome==1 & boot.cloned.df$freq>0,], #exclude freq=0

weights = freq, family=quasibinomial()))

#change name of clone to A

names(bcoef)[5] <- "A"

names(wcoef)[5] <- "A"

#4. Expand rows according to freq (uncloned)

exp.df <- bootdf[bootdf$t==0 & bootdf$touseweight==1 & bootdf$freq > 0,] #get baseline from sampled only

exp.df <- exp.df[rep(seq(nrow(exp.df)), exp.df$freq),]

#nrow(exp.df) == 717 #expanded should go back to 717 rows

#5. Create baseline dataset from expanded (uncloned)

baseline <- exp.df[exp.df$t==0 & exp.df$touseweight==1, c('age_t0', 'sex', 're_black', 're_hisp', 're_other', 'gfr0', 'chol0', 'dm_yn0', 'htn_yn0', 'comp_comorb_yn0', "ins_pri", "ins_gov", "yod_centered")]

#5. set up dfSetA0 and dfSetA1

t <- 0:maxT

temp <- as.data.frame(rcspline.eval(t, knots=c(3,9,15,21), inclx = TRUE))

names(temp) <- c("t_rcs1", "t_rcs2", "t_rcs3")

dfSetA0 <- data.frame(t_rcs1 = temp$t_rcs1, t_rcs2 = temp$t_rcs2, t_rcs3 = temp$t_rcs3, A=0)

dfSetA1 <- data.frame(t_rcs1 = temp$t_rcs1, t_rcs2 = temp$t_rcs2, t_rcs3 = temp$t_rcs3, A=1)

#6. Run MC simulation in bootstrapped data

nind <- nrow(exp.df)

margHRb <- MCsimHR(i=i, Nsim = 100000, baseline, nind=nind, maxT=60, coeffit=bcoef, dfSetA0, dfSetA1)

tvmargHRb <- MCsimHR(i=i, Nsim = 100000, baseline, nind=nind, maxT=60, coeffit=wcoef, dfSetA0, dfSetA1)

# # Return data frame

return(data.frame(baseA = margHRb, tvA = tvmargHRb)) #tvA = tvmargHRb

}

saveRDS(mhr.bootres.test2, "target - marg hr results - December 2021.RDS")

summary(mhr.bootres.test2)
