# Supplementary material for: Designing target trials using electronic health records: A case study of second-line disease-modifying anti-rheumatic drugs and cardiovascular disease outcomes in patients with rheumatoid arthritis
Source: PLoS One. 2024 Jun 14;19(6):e0305467. doi: 10.1371/journal.pone.0305467 (PMC11178161; doi:10.1371/journal.pone.0305467)
Supplement: S1 Table — (DOCX) [file pone.0305467.s006.docx]

**S1 Table.** **ICD codes for different conditions.**

| Condition | Definition |
| --- | --- |
| 1. **Rheumatoid Arthritis** | |
| Rheumatoid Arthritis* | At least 2 diagnosis codes within a 2-year period (same code twice in a 2-year period counts); codes include ICD-9 714.0, 714.2, ICD-10 M05.xx, M06.xx |
| 1. **Outcomes** | |
| Myocardial infarction | At least 2 diagnosis codes within a 2-year period (same code twice in a 2-year period counts), and the first of the 2 codes occurring prior to time zero.  ICD9: 41000, 41001, 41002, 41010, 41011, 41012, 41020, 41021, 41022, 41030, 41031, 41032, 41040, 41041, 41042, 41050, 41051, 41052, 41060, 41061, 41062, 41070, 41071, 41072, 41080, 41081, 41082, 41090, 41091, 41092, 412  ICD10: I2101, I2102, I2109, I2111, I2119, I2121, I2129, I213, I214, I220, I221, I222, I228, I229, I252 |
| Heart Failure | At least 2 diagnosis codes within a 2-year period (same code twice in a 2-year period counts), and the first of the 2 codes occurring prior to time zero; codes include 398.91, 401.01, 402.01, 402.11, 402.91, 404.03, 404.11, 404.13, 404.91, 404.93, 428.xx |
| Stroke | At least 1 diagnosis code for ICD9-CM 433-436; ICD10-CM I60-I63 |
| 1. **Conditions for exclusion** | |
| Baseline chronic ischemic heart disease, coronary artery disease | At least 2 diagnosis codes within a 2-year period (same code twice in a 2-year period counts), and the first of the 2 codes occurring prior to time zero.  ICD9: 4110, 4111, 41181, 41189, 4130, 4139, 41400, 41401, 41402, 41403, 41404, 41405, 41406, 41407, 4142, 4143, 4144, 4148, 4149  ICD10: I200, I208, I209, I240, I241, I248, I249, I2510, I25110, I25111, I25118, I25119, I255, I256, I25700, I25701, I25708, I25709, I25710, I25711, I25718, I25719, I25720, I25721, I25728, I25729, I25730, I25731, I25738, I25739, I25750, I25751, I25758, I25759, I25760, I25761, I25768, I25769, I25790, I25791, I25798, I25799, I25810, I25811, I25812, I2582, I2583, I2584, I2589, I259 |
| Coronary Artery Bypass | At least 2 diagnosis codes within a 2-year period (same code twice in a 2-year period counts), and the first of the 2 codes occurring prior to time zero.  ICD9 Procedure code: 3610, 3611, 3612, 3613, 3614, 3615, 3616, 3617, 3619  ICD10 Procedure code: 0210093, 0210098, 0210099, 021009C, 021009F, 021009W, 02100A3, 02100A8, 02100A9, 02100AC, 02100AF, 02100AW, 02100J3, 02100J8, 02100J9, 02100JC, 02100JF, 02100JW, 02100K3, 02100K8, 02100K9, 02100KC, 02100KF, 02100KW, 02100Z3, 02100Z8, 02100Z9, 02100ZC, 02100ZF, 0210344, 02103D4, 0210444, 0210493, 0210498, 0210499, 021049C, 021049F, 021049W, 02104A3, 02104A8, 02104A9, 02104AC, 02104AF, 02104AW, 02104D4, 02104J3, 02104J8, 02104J9, 02104JC, 02104JF, 02104JW, 02104K3, 02104K8, 02104K9, 02104KC, 02104KF, 02104KW, 02104Z3, 02104Z8, 02104Z9, 02104ZC, 02104ZF, 0211093, 0211098, 0211099, 021109C, 021109F, 021109W, 02110A3, 02110A8, 02110A9, 02110AC, 02110AF, 02110AW, 02110J3, 02110J8, 02110J9, 02110JC, 02110JF, 02110JW, 02110K3, 02110K8, 02110K9, 02110KC, 02110KF, 02110KW, 02110Z3, 02110Z8, 02110Z9, 02110ZC, 02110ZF, 0211344, 02113D4, 0211444, 0211493, 0211498, 0211499, 021149C, 021149F, 021149W, 02114A3, 02114A8, 02114A9, 02114AC, 02114AF, 02114AW, 02114D4, 02114J3, 02114J8, 02114J9, 02114JC, 02114JF, 02114JW, 02114K3, 02114K8, 02114K9, 02114KC, 02114KF, 02114KW, 02114Z3, 02114Z8, 02114Z9, 02114ZC, 02114ZF, 0212093, 0212098, 0212099, 021209C, 021209F, 021209W, 02120A3, 02120A8, 02120A9, 02120AC, 02120AF, 02120AW, 02120J3, 02120J8, 02120J9, 02120JC, 02120JF, 02120JW, 02120K3, 02120K8, 02120K9, 02120KC, 02120KF, 02120KW, 02120Z3, 02120Z8, 02120Z9, 02120ZC, 02120ZF, 0212344, 02123D4, 0212444, 0212493, 0212498, 0212499, 021249C, 021249F, 021249W, 02124A3, 02124A8, 02124A9, 02124AC, 02124AF, 02124AW, 02124D4, 02124J3, 02124J8, 02124J9, 02124JC, 02124JF, 02124JW, 02124K3, 02124K8, 02124K9, 02124KC, 02124KF, 02124KW, 02124Z3, 02124Z8, 02124Z9, 02124ZC, 02124ZF, 0213093, 0213098, 0213099, 021309C, 021309F, 021309W, 02130A3, 02130A8, 02130A9, 02130AC, 02130AF, 02130AW, 02130J3, 02130J8, 02130J9, 02130JC, 02130JF, 02130JW, 02130K3, 02130K8, 02130K9, 02130KC, 02130KF, 02130KW, 02130Z3, 02130Z8, 02130Z9, 02130ZC, 02130ZF, 0213344, 02133D4, 0213444, 0213493, 0213498, 0213499, 021349C, 021349F, 021349W, 02134A3, 02134A8, 02134A9, 02134AC, 02134AF, 02134AW, 02134D4, 02134J3, 02134J8, 02134J9, 02134JC, 02134JF, 02134JW, 02134K3, 02134K8, 02134K9, 02134KC, 02134KF, 02134KW, 02134Z3, 02134Z8, 02134Z9, 02134ZC, 02134ZF  CPT codes: 33510, 33511, 33512, 33513, 33514, 33516, 33517, 33518, 33519, 33521, 33522, 33523, 33533, 33534, 33535, 33536 |
| Prior Percutaneous Coronary Intervention | At least 2 diagnosis codes within a 2-year period (same code twice in a 2-year period counts), and the first of the 2 codes occurring prior to time zero.  ICD9 Procedure code: 0066, 1755, 3601, 3602, 3605, 3606, 3607, 3609  ICD 10 Procedure code: 0270046, 027004Z, 02700D6, 02700DZ, 02700T6, 02700TZ, 02700Z6, 02700ZZ, 0270346, 027034Z, 02703D6, 02703DZ, 02703T6, 02703TZ, 02703Z6, 02703ZZ, 0270446, 027044Z, 02704D6, 02704DZ, 02704T6, 02704TZ, 02704Z6, 02704ZZ, 0271046, 027104Z, 02710D6, 02710DZ, 02710T6, 02710TZ, 02710Z6, 02710ZZ, 0271346, 027134Z, 02713D6, 02713DZ, 02713T6, 02713TZ, 02713Z6, 02713ZZ, 0271446, 027144Z, 02714D6, 02714DZ, 02714T6, 02714TZ, 02714Z6, 02714ZZ, 0272046, 027204Z, 02720D6, 02720DZ, 02720T6, 02720TZ, 02720Z6, 02720ZZ, 0272346, 027234Z, 02723D6, 02723DZ, 02723T6, 02723TZ, 02723Z6, 02723ZZ, 0272446, 027244Z, 02724D6, 02724DZ, 02724T6, 02724TZ, 02724Z6, 02724ZZ, 0273046, 027304Z, 02730D6, 02730DZ, 02730T6, 02730TZ, 02730Z6, 02730ZZ, 0273346, 027334Z, 02733D6, 02733DZ, 02733T6, 02733TZ, 02733Z6, 02733ZZ, 0273446, 027344Z, 02734D6, 02734DZ, 02734T6, 02734TZ, 02734Z6, 02734ZZ, 02C03ZZ, 02C04ZZ, 02C13ZZ, 02C14ZZ, 02C23ZZ, 02C24ZZ, 02C33ZZ, 02C34ZZ, X2C0361, X2C1361  CPT codes: 92920, 92921, 92924, 92925, 92928, 92929, 92933, 92934, 92937, 92938, 92941, 92943, 92944, 92980, 92981, 92982, 92984, 92995, 92996, C9600, C9601, C9602, C9603, C9604, C9605, C9606, C9607, C9608, G0290, G0291 |
| Baseline chronic ischemic heart disease, coronary artery disease | At least 2 diagnosis codes within a 2-year period (same code twice in a 2-year period counts), and the first of the 2 codes occurring prior to time zero.  ICD9 Code: 4110, 4111, 41181, 41189, 4130, 4139, 41400, 41401, 41402, 41403, 41404, 41405, 41406, 41407, 4142, 4143, 4144, 4148, 4149  ICD10 Code: I200, I208, I209, I240, I241, I248, I249, I2510, I25110, I25111, I25118, I25119, I255, I256, I25700, I25701, I25708, I25709, I25710, I25711, I25718, I25719, I25720, I25721, I25728, I25729, I25730, I25731, I25738, I25739, I25750, I25751, I25758, I25759, I25760, I25761, I25768, I25769, I25790, I25791, I25798, I25799, I25810, I25811, I25812, I2582, I2583, I2584, I2589, I259 |
| Cancer (all cancer excluding non-melanoma skin cancer) | At least 2 diagnosis codes within a 2-year period (same code twice in a 2 year period counts); ICD9 140–209 *excluding 173*, ICD-10 C00-C97 *excluding C44* |
| Psoriasis | At least 2 diagnosis codes within a 2-year period (same code twice in a 2 year period counts); codes include ICD-9 696.1, ICD10 L40.xx |
| Systematic lupus erythematosus | At least 3 diagnosis codes (repeat of the same code OK) occurring in 3 separate months; codes are: ICD-9 710.0, ICD-10 M32.1X, M32.8, M32.9 |
| Crohns | At least 2 diagnosis codes within a 2-year period (same code twice in a 2 year period counts); codes include ICD-9 555.XX, ICD-10 K50.XX |
| Ulcerative colitis | At least 2 diagnosis codes within a 2-year period (same code twice in a 2 year period counts); codes include ICD-9 556.XX, ICD-10 K51.XX |
| Systemic sclerosis | At least 2 diagnosis codes within a 2-year period (same code twice in a 2 year period counts); codes include ICD-9 710.1, ICD-10 M34.xx |
| Dermatomyositis | At least 2 diagnosis codes within a 2-year period (same code twice in a 2 year period counts); codes include ICD-9 710.3, ICD-10 M33.0x, M33.1x, M33.9x |
| Polymyositis | At least 2 diagnosis codes within a 2-year period (same code twice in a 2 year period counts); codes include ICD-9 710.4, ICD-10 M33.2x |
| Atopic dermatitis | At least 2 diagnosis codes within a 2-year period (same code twice in a 2 year period counts); codes include ICD-9 691.8, ICD-10 L20.9 |
| Hepatitis B | At least 1 diagnosis code of ICD-9 070.41, 070.44, 070.51, 070.54, or V02.62, ICD-10 B17.1, B18.2, B19.2 |
| Hepatitis C | At least 1 diagnosis code of ICD-9 070.2X, 070.3X, V02.61, ICD-10 B17.0, B18.0, B18.1, B19.1 |
| Tuberculosis | At least 2 diagnosis codes within a 2-year period (same code twice in a 2 year period counts); ICD-9 010.x - 018.x, ICD-10 A15-A19 |
| HIV | Lab-based definition same as from most recent Feinstein HIV cohort:   - Positive HIV-1 antibody or serology,  OR - Positive (>0) HIV viral load,  OR   At least 3 orders of HIV viral load and CD4 T cell count ordered on the same day as any HIV viral load results |
| 1. **Condition for adjustment** | |
| Hypertension | At least 2 diagnosis codes within a 2-year period (same code twice in a 2-year period counts), and the first of the 2 codes occurring prior to time zero OR 1 diagnosis code of hypertension on a hospital discharge abstract record.  Codes included are:   - ICD9: 405.99, 405, 405.9, 401, 401.1, 401.0, 405.91, 405.0, 401.9 - ICD10: I10, I15, I15.xx |
| Diabetes Mellitus | At least 2 diagnosis codes within a 2-year period (same code twice in a 2-year period counts), and the first of the 2 codes occurring prior to time zero:  ICD9: 250, 250, 250, 250.01, 250.01AK, 250.01C, 250.02, 250.03, 250.1, 250.1, 250.11, 250.12, 250.13, 250.2, 250.2, 250.21, 250.22, 250.23, 250.3, 250.3, 250.31, 250.32, 250.33, 250.4, 250.4, 250.41, 250.42, 250.43, 250.5, 250.5, 250.51, 250.52, 250.53, 250.6, 250.6, 250.61, 250.62, 250.63, 250.7, 250.7, 250.7, 250.7, 250.71, 250.71, 250.72, 250.72, 250.73, 250.73, 250.8, 250.8, 250.81, 250.82, 250.83, 250.9, 250.9, 250.91, 250.92, 250.93, 250.99  ICD10: E10.0, E10.10, E10.21, E10.22, E10.29, E10.311, E10.319, E10.321, E10.329, E10.331, E10.339, E10.341, E10.349, E10.351, E10.359, E10.36, E10.39, E10.40, E10.41, E10.42, E10.43, E10.49, E10.51, E10.52, E10.59, E10.610 E10.618 E10.621, E10.649, E10.65, E10.69, E10.7, E10.8, E10.9, E11.00, E11.01, E11.21, E11.22, E11.29, E11.311, E11.319, E11.321, E11.329, E11.331, E11.339, E11.341, E11.349, E11.351, E11.359, E11.36, E11.39, E11.40, E11.41, E11.42, E11.43, E11.44, E11.49, E11.51, E11.52, E11.59, E11.610, E11.618, E11.620, E11.621, E11.622, E11.628, E11.638, E11.641, E11.649, E11.65, E11.69, E11.8, E11.9, E13.00, E13.01, E13.10, E13.11, E13.21, E13.22, E13.29, E13.319, E13.329, E13.339, E13.341, E13.359, E13.39, E13.40, E13.42, E13.43, E13.51, E13.52, E13.59, E13.610, E13.621, E13.628, E13.630, E13.649, E13.65, E13.69, E13.8, E13.9  AND has EITHER:  (1) any Hemoglobin A1c value of 6.5% or higher; OR (2) any antidiabetic medication. |
| Atrial fibrillation or flutter | At least 2 diagnosis codes within a 2-year period (same code twice in a 2-year period counts), and the first of the 2 codes occurring prior to time zero.  ICD9-CM 427.3, (including 427.31, 427.32); ICD10-CM I48; CPT codes 93653, 93655, 93656, 93657  OR  ECG Report matching:  REGEXP_LIKE(IMPRESSION,'((^\|[ \.])AF($\|[ \.]))\|(a\w*[ .]?(fib[\w]*\|flutter))','i')  This should match:  Afib  A* fib*  Aflutter  A* flutter |
| Chronic Obstructive Pulmonary Disease | ICD-9 or 10 codes: 491, 491, 491.1, 491.2, 491.2, 491.21, 491.22, 491.8, 491.9, 492, 492, 492.8, 496, 496, 496.11, 496.2, J41.0, J41.1, J41.8, J42, J43.0, J43.1, J43.2, J43.8, J43.9, J44.0, J44.1, J44.9 |
| Chronic Kidney Disease | ICD-10CM: N181, N182, N183, N184, N185, N186, N189, N19 |

*Reference for RA ICD code: Huang S, Huang J, Cai T, Dahal KP, Cagan A, He Z, Stratton J, Gorelik I, Hong C, Cai T, Liao KP. Impact of ICD10 and secular changes on electronic medical record rheumatoid arthritis algorithms. Rheumatology. 2020 Dec;59(12):3759-66.
