# Supplementary material for: Designing target trials using electronic health records: A case study of second-line disease-modifying anti-rheumatic drugs and cardiovascular disease outcomes in patients with rheumatoid arthritis
Source: PLoS One. 2024 Jun 14;19(6):e0305467. doi: 10.1371/journal.pone.0305467 (PMC11178161; doi:10.1371/journal.pone.0305467)
Supplement: S2 Table — (DOCX) [file pone.0305467.s007.docx]

**S2 Table.** **Types of First DMARD started during Grace Period and Average Time to Starting DMARD (n=289), Northwestern Medicine, January 2000 to June 2020.**

| DMARD | Type | Mechanism of action | n (%) | Mean Month to start (SD) |
| --- | --- | --- | --- | --- |
| Abatacept | Biologic | CD80/86 inhibitor | 7 (2.4) | 6.49 (2.33) |
| Adalimumab | Biologic | Tumor necrosis factor inhibitor | 71 (24.6) | 6.69 (5.85) |
| Etanercept | Biologic | Tumor necrosis factor inhibitor | 45 (15.6) | 5.36 (2.76) |
| Hydroxychloroquine | Conventional | intracellular toll-like receptor TLR9 inhibitor | 62 (21.5) | 7.42 (10.46) |
| Infliximab | Biologic | Tumor necrosis factor inhibitor | 18 (6.2) | 6.03 (7.03) |
| Leflunomide | Conventional | dihydroorotate dehydrogenase inhibitor | 33 (11.4) | 7.05 (4.01) |
| Sulfasalazine | Conventional | oxidative, nitrative, and nitrosative damage prevention | 23 (8.0) | 7.20 (4.99) |
| Tofacitinib | Targeted synthetic | Janus kinase inhibitor | 13 (4.5) | 4.51 (5.72) |
| Others* | Mixed | Mixed | 17 (5.9) | 7.03 (8.77) |

Note: Abbreviations: DMARD - Disease-modifying Anti-rheumatic Drugs, SD – standard deviation. *Others include conventional (azathioprine, minocycline), biologic (certolizumab, golimumab, rituximab, tocilizumab) and targeted synthetic (upadacitnib) DMARDs. Data query also included searching for prescription of Baricitinib, Belatacept (not usually used for rheumatoid arthritis), Belimumab (not usually used for rheumatoid arthritis), and Sarilumab but these did not end up as the additional DMARD in the sample. Anakinra was not included in the data query.
