# Supplementary material for: Designing target trials using electronic health records: A case study of second-line disease-modifying anti-rheumatic drugs and cardiovascular disease outcomes in patients with rheumatoid arthritis
Source: PLoS One. 2024 Jun 14;19(6):e0305467. doi: 10.1371/journal.pone.0305467 (PMC11178161; doi:10.1371/journal.pone.0305467)
Supplement: Attachment — Submitted filename: target trial - plos one - response to v2 comments 08may2024.docx [file pone.0305467.s008.docx]

**Detailed Responses to Reviewer Comments for PONE-D-23-36062_R1:**

**Comment 1:** My primary concern is whether the proposed model has been validated, such as through simulation. What are the benefits gained compared to other approaches? Specifically, I am wondering how much bias will be reduced compared to a simple non-weighted model.

Response: We have edited the introduction, methods, and discussion section of the paper to stress that the advantages of this approach does not lie on the statistical analysis alone. Specifically, the TT approach ensures that the researcher properly specifies the intervention under study. For our research question, we are specifically concerned about the inappropriate practice of comparing ever vs never DMARD users when faced with a scenario where treatment can occur at multiple time points. We highlight Danaei 2011’s (doi: 10.1177/0962280211403603) point that this comparison leads to “a gamble in which the investigators bet that the amount of selection bias introduced is less than the amount of confounding eliminated.” Instead of taking this gamble, our elaborate approach allows handling of both selection and confounding.

To answer the concerns of the validity of the statistical estimation approach (weighted pooled logistic regression), we add details about related simulations which assessed the performance of statistical estimation approaches that can be used in this setting. We think simulation is outside the scope of this paper and the references adequately support the validity of the model we used in the paper. We think a non-weighted model is inconsistent with the various biases we needed to account for especially with the introduction of a grace period. However, we have conducted a naïve analysis which compares ever vs never users to see how much selection bias may have influenced the findings.

Changes in text:

Introduction: “In this case, the Food and Drug Administration has encouraged the addition of observational CER studies to post-market safety evidence;[17] the methods described here can be generalized to comparisons of therapies that are confounded by treatment due to indication. This approach can also be used in scenarios where treatment can be initiated at multiple time points and serve as a more principled alternative to the commonly utilized approach of comparing never to ever initiators.”

Methods: “Unlike RCTs where points of randomization are clear and specified in advance, individuals in EHRs do not necessarily share the same timing of treatment initiation/discontinuation. Comparing never to ever DMARD users based on the full data without specifying timing of initiation, induces selection and leads to an unclear causal question. However, using a too strict definitions such as ‘started additional DMARD exactly after 8 weeks of MTX use’ would lead to incredibly small sample sizes.”

Discussion: “Oftentimes, researchers would do a naïve analysis which compares ever versus never treated (e.g., ever used DMARD vs never used DMARD) by using future events to assign exposure status. This approach forces the researcher to make “a gamble in which the investigators bet that the amount of selection bias introduced is less than the amount of confounding eliminated.”[7] Based on our analysis, the selection induced by doing a naïve comparison might not have altered the overall conclusion. Still, the point estimate of the naïve analysis is further away from the null compared to the emulated value (naïve HR: 0.62 vs emulated OR: 0.71). Importantly, there is a more fundamental issue with the naïve analysis: the comparison answers an unclear question that cannot be applied in real life: How can one ensure a person never uses a drug? How can one ask a person to initiate a drug but not specify when to initiate it? Our paper illustrates an alternative to this problematic naïve approach through design (e.g., introducing grace periods) and analysis (e.g., cloning and re-weighting).

We again stress that the TT emulation framework is not prescriptive in terms of statistical estimation. Depending on the question and data, even the commonly used linear regression model with covariate adjustment may suffice. For our question and data, we used grace periods with weighted pooled logistic regression. Pooled logistic regression has been shown perform comparably with time-dependent Cox models especially in rare outcomes.[48] Use of weights to account for post-baseline confounding have been shown to obtain unbiased estimates in simulation of trial data with null effects.[49] We could have also used approaches like marginal structural modelling, longitudinal matching or sequential nested trials to overcome the challenges of time-varying exposures although it would necessitate modifying the question being answered. (e.g., matching produces average treatment among the treated).[50,51]”

**Comment 2:** A series of sensitivity analyses were conducted, revealing that some estimates closely align while others do not. Has a model selection procedure been employed? What conclusions can be drawn from these diverse results, and how should they be interpreted? What recommendations emerge from this analysis?

Response: We revised the paper to clarify that we conducted theory-based variable selection with the assistance of directed acyclic graphs. This is the recommended approach instead of using data driven covariate selection approaches (e.g., lasso regression).(see Staerk and Mayr 2024, doi.org/10.1093/aje/kwad193) We have also modified the discussion to state more clearly what can be concluded from the sensitivity analysis and the implications of this work for research practice.

Changes:

Methods: “The resulting model (Step 3) can then be used to calculate marginal survival curves, risk differences at select times, 5-year mean restricted survival time, and the average hazards ratio over follow-up. Covariates selected to be included in the various models should be guided by existing knowledge or theory and by constructed directed acyclic graphs.[37–39] Researchers may opt to use data-driven approaches for covariate selection (e.g., lasso) but it can add to the computational time and complexity. Covariates can be used in both weighting (steps 1 and 2) and outcome models (step 3) as it may safeguard against residual imbalance.[40]”

Discussion: “Here, we did not observe evidence of differences in MACE risk, a finding that is limited by the small number of outcomes observed. This finding was robust to design-based and statistical choices such as grace period length and functional form of time to specify baseline hazard.”

**Comment 3:** Table 3. Why the two groups were presented at 24m? Why not at baseline for baseline characteristics?

Response: We appreciate this concern. We do present baseline characteristics for the overall sample in Table 3. However, at baseline treatment assignment is not finalized – individuals may be compatible with more than one treatment arm as we are comparing treatment initiation by the end of the grace period, which ends after study baseline. So, instead we conducted the comparison of covariates at the end of the grace period since it is only at this point where the exposure assignment is firmly assigned.

Changes:

“At the month 24 (end of grace period and point where exposure assignment was finalized), those in the MTX+DMARD group (n = 287) were younger, had a lower proportion of White patients, a higher proportion of Hispanic patients, had a higher proportion with DM and HTN, and had higher eGFR compared to those in the MTX monotherapy (n=352). (Table 3)”

**Comment 4:** Table 4. Can the comorbidity status be used in the weighting and in the model twice time?

Response: To answer the reviewer’s query, including the same covariate in both the weighting and outcome model is accepted practice. It is one way to address any residual imbalance that is incompletely addressed by weighting. We added this detail and the appropriate citation in the text.

Changes:

“Covariates can be used in both weighting (steps 1 and 2) and outcome models (step 3) as it may safeguard against residual imbalance.[40] All weights should be stabilized (and possibly truncated) to prevent large weights on rare individuals.”
